# Structure–Piezoelectric Property Relationships of Thin Films Composed of Electrospun Aligned Poly(vinylidene fluoride) Nanofibers

**DOI:** 10.3390/nano14060491

**Published:** 2024-03-08

**Authors:** Priangga Perdana Putra, Shuichi Akasaka, Yuichi Konosu, Shaoling Zhang, Akihiko Tanioka, Hidetoshi Matsumoto

**Affiliations:** Department of Materials Science and Engineering, School of Materials and Chemical Technology, Tokyo Institute of Technology, 2-12-1 Ookayama, Meguro-ku, Tokyo 152-8552, Japan

**Keywords:** poly(vinylidene fluoride), nanofiber, electrospinning, crystal orientation, piezoelectric property

## Abstract

In the past two decades, many studies on piezoelectric nanofibers (NFs) prepared from poly(vinylidene fluoride) (PVDF) and its copolymers, including single NFs, randomly oriented nonwoven mats, and aligned NFs, have been reported. However, studies on the relationships between the PVDF NF diameter, the orientation of the β-phase crystals inside NFs, and the piezoelectric properties of the NFs are still limited. In this study, the effect of the fiber diameter on the internal molecular packing/orientation and piezoelectric properties of aligned PVDF NF thin films was investigated. Herein, piezoelectric thin films composed of densely packed, uniaxially aligned, PVDF NFs with diameters ranging from 228 to 1315 nm were prepared by means of electrospinning with a rotating collector and successive hot-pressing and poling. The effect of the diameters of PVDF NFs on their internal structures, as well as the piezoelectric properties of the thin films, was investigated. All prepared NFs mainly contained β-phase crystals with a similar total crystallinity. The orientation of the β-phase crystals inside the NFs increased with an increase in the fiber diameter, resulting in an improved transverse piezoelectric coefficient (*d*_31_) for the thin films. The output voltage of the prepared thin films reached a maximum of 2.7 V at 104 Hz.

## 1. Introduction

Recently, the rapid growth of fifth-generation (5G) mobile networks and the Internet of Things (IoT) has triggered an interest towards small electronics such as sensors, actuators, wireless transmitters, and energy harvesters for health monitoring, biochemical detection, environmental protection, remote control, wireless transmission, and security [1]. Because of the potential for sensing minute displacements, harvesting mechanical energy, and powering low-power-consumption sensors, piezoelectricity, which comes from the Greek word *piece*, corresponding to press, has garnered a lot of attention. In fact, piezoelectric devices, which can transform mechanical energy into electricity and vice versa, are key components utilized for detecting external stimuli [2] as well as for scavenging small mechanical energies (e.g., ambient vibration and human motions) [3].

Piezoelectric materials are polar dielectric materials that generate an electric field when subjected to mechanical deformation. Inorganic piezoelectric materials were the first piezoelectric materials. Various inorganic materials, such as wurtzite materials (e.g., ZnO and GaN), zinc-blende materials, and perovskite materials (e.g., Pb(Zr_x_Ti_1−x_)O_3_ or PZT and BaTiO_3_ or BTO), have shown excellent piezoelectric effects [1,2]. In comparison to inorganic materials, organic polymers have attracted much attention because of their other advantages, such as being lightweight and flexible, and because they have low acoustic impedance, low mechanical impedance, and low-temperature processability [3].

Among piezoelectric polymers, PVDF is the most widely used. Kawai first reported a piezoelectric effect in poly(vinylidene fluoride) (PVDF) in 1969 [4]. PVDF and its copolymers exhibit relatively higher piezoelectric coefficients compared to other piezoelectric polymers, as well as better mechanical flexibility, chemical stability, and biocompatibility [5]. PVDF, a semicrystalline polymer composed of repeating CF_2_-CH_2_ units, can exhibit various crystalline phases, including α, β, γ, δ, and ε phases, based on the chain conformation as trans or gauche linkages. Of these, the β-phase, with an all-trans planar zigzag chain conformation (TTTT), shows the highest piezoelectric activity [2,6]. An effective method to form β-phase crystals is electrospinning [7,8], which is based on an electrohydrodynamic process for the formation of continuous thin fibers [9,10]. Electrospinning has been used to obtain piezoelectric polymer nanofibers (NFs) with polymer chains or helices oriented parallel to the fiber axis (e.g., PVDF [8,11], poly(γ-benzyl α,_L_-gultamate) [12], and polyamide 11 [13,14]). A high molecular orientation along the fiber axis is induced by extremely high draw ratios and strain rates during the spinning process (note that the β-phase crystals in PVDF NFs can also be formed by means of blow spinning without an electric field [15]). In the past two decades, many studies on piezoelectric NFs prepared from PVDF and its copolymers, including single NFs, randomly oriented NF nonwoven mats, and aligned NFs, have been reported [16,17,18,19,20,21,22,23]. To improve the piezoelectric performance of PVDF NFs, strong electric fields (e.g., near-field electrospinning [24]) and the addition of piezoelectric inorganic materials [25] are commonly used in addition to the optimization of the spinning solution and conditions [21,26]. Such improvements allow us to monitor small body vibration signals (e.g., for speech recognition, respiration monitoring, and pulse monitoring) as well as large body motion signals (e.g., gait monitoring, neck flexion, elbow flexion, and knee flexion) [2]. However, studies on the relationships between the PVDF NF diameter, orientation of the β-phase crystals within NFs, and the piezoelectric properties of the NFs, including single NFs, randomly oriented NF nonwoven mats, and aligned NFs, are still limited. One reason is that directly characterizing the crystal orientation and/or piezoelectric coefficients of the NF samples is not easy, particularly for an NF assembly such as nonwoven mats [27,28].

Herein, in order to clarify the structure–piezoelectric property relationships of PVDF NFs, particularly focusing on the effect of the internal molecular packing/orientation within NFs (i.e., the orientation of β-phase crystals) on their piezoelectric properties, we prepared aligned PVDF NF thin films instead of randomly oriented nonwoven mats. In the electrospinning technique, the NFs deposited on the flat-plate collector provides randomly oriented NF nonwoven mats, but the rotating collector is widely used to collect aligned fibers by controlling the rotating speed of the collector tuned with the velocity of liquid jet ejected from the spinneret during spinning [29]. Aside from the rotating collector, a collector composed of two conductive plates is another approach for preparing aligned NFs [29]. Kang et al. reported that the aligned PVDF NFs exhibited improved crystallinity and a higher content of β-phase crystals compared to randomly oriented nonwoven mats, consequently improving the piezoelectric response [30]. Thus, aligned arrays of PVDF NFs generally show a high density and preferential orientation, leading to superior piezoelectric responses with mechanical robustness [23]. More recently, aligned PVDF NF-based piezoelectric nanogenerators (PENGs) [31] and highly aligned PVDF NF arrays integrated with electronic skin sensors have been developed [32]. We believe that the alignment of electrospun PVDF NFs plays an important role in the (semi)quantitative analysis of the crystal orientation within NFs using X-ray diffraction (XRD) and results in an enhancement in the piezoelectric performance. We adapted a rotating collector to efficiently prepare thicker aligned NF samples.

In this study, we prepared densely packed, uniaxially aligned, PVDF NF thin films with different diameters by means of electrospinning with a rotating collector, followed by hot-pressing and poling, and then characterized their β-phase crystal orientation and transverse piezoelectric coefficient (*d*_31_). XRD and differential scanning calorimetry (DSC) were used to characterize the crystal form, crystal orientation, and degree of crystallinity within the NFs. The aim of this study was to investigate the relationship between the fiber diameters of electrospun PVDF NFs, their internal structures (i.e., crystalline phase and orientation), and the piezoelectric properties of the aligned PVDF NF thin films. To the best of our knowledge, this is the first report on the relationship between the diameter of aligned PVDF NFs, their internal β-phase crystal orientation, and their piezoelectric properties.

## 2. Materials and Methods

### 2.1. Materials

Poly(vinylidene fluoride) (PVDF; *M*_w_ = 275,000 g/mol) was purchased from Sigma-Aldrich (St. Louis, MO, USA). *N*,*N*-Dimethylacetamide (DMAc; purity, ≥99%) and acetone (purity, ≥99%) were purchased from Fujifilm Wako Pure Chemicals, Osaka, Japan. The reagents were used as received without further purification.

### 2.2. Preparation of Nanofibers

In electrospinning, the fiber diameter depends on the solution’s properties (e.g., viscosity, boiling point, electrical conductivity, surface tension, and permittivity) and/or operating conditions (e.g., applied voltage, nozzle-to-spinneret distance, and flow rate). Among them, the viscosity and boiling point of the spinning solutions are crucial factors for controlling the fiber diameter. To prepare the spinning solutions, PVDF pellets were dissolved in DMAc or a DMAc/acetone mixture (1:2 *v*/*v*) at different concentrations (20 to 28 wt%). The solutions were stirred at 80 °C (for DMAc) or at 50 °C (for DMAc/acetone mixture) for at least 3 h. The viscosity of the spinning solutions was measured using a torsional oscillation-type viscometer (VM-100A, CBC Materials, Tokyo, Japan) at approximately 25 °C. The compositions and viscosities of the spinning solutions are listed in Table 1.

**Figure 1:**
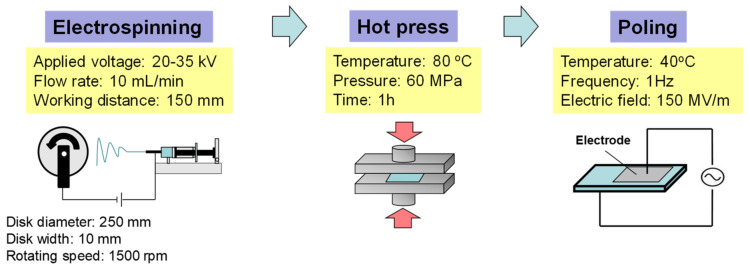
Schematic diagram of electrospinning and successive hot-pressing and poling for preparation of piezoelectric NF thin films.

The electrospinning setup (Figure 1) was the same as that used in our previous study [33]. The spinning solution was placed in a syringe with a stainless steel nozzle (inner diameter: 0.51 mm) as the spinneret. The nozzle was connected to a high-voltage direct current power supply (HAR-100P0.3, Matsusada Precision, Otsu, Japan). A constant-volume flow rate was maintained using a syringe infusion pump (MCIP-III, Minato Concept, Tokyo, Japan). A rotating disk (disk diameter: 250 mm; width: 10 mm) was used as the collector to align the electrospun NFs. Cu tape was used as the substrate and placed on the periphery of the rotating disk. The applied voltage was 20–35 kV, the distance between the nozzle tip and collector was 150 mm, the flow rate was 10 μL/min, and the rotating speed of the collector was 1500 rpm. All electrospinning was carried out at approximately 25 ± 1 °C and under a relative humidity of less than 30%. Freestanding thin films composed of the aligned NFs were obtained after deposition.

### 2.3. Hot-Pressing and Poling

The as-spun aligned NF thin films on the Cu tape substrate were hot-pressed using a compact hot-press apparatus (Imoto Machinery, Kyoto, Japan) at 80 °C under a pressure of 60 MPa for 1 h (Figure 1). The hot-pressing was carried out in order to remove the spaces between the NFs, which could induce discharge during poling. Teflon^®^ sheets and aluminum plates were used as the mold release films and spacers, respectively. The hot-pressed NF thin films were then poled using an AC electric field. The amplitude of the electric field was increased over several cycles to a maximum field of 150 MV/m at 1 Hz of the sweeping frequency in a silicone oil bath at 40 °C. Then, the electric field was stopped when it reached zero. Note that the applied electric field was higher than 150 MV/m, resulting in discharge of the NF thin films. The sputtered Au layers on both surfaces of the sample were used for the electrode (Figure 1). After poling, the NF thin films were washed sufficiently with ethanol and vacuum-dried for 3 h. The thin film thickness was determined using a height gauge (Digimatic Indicator ID-C112AXB, Mitutoyo, Kawasaki, Japan).

### 2.4. Characterization

The morphologies of the prepared aligned NFs and NF thin films were observed using a scanning electron microscope (SEM, SM-200, Topcon, Tokyo, Japan) operated at 10 kV for the accelerated voltage of electrons. All of the samples were sputter-coated with Au. The average fiber diameters were determined by means of SEM image analysis using the ImageJ software (Ver. 1.53, National Institutes of Health, Bethesda, MD, USA). DSC measurements of prepared NF thin films were performed using a calorimeter (DSC 6100, Seiko Instruments, Chiba, Japan) under a N_2_ atmosphere. The heating rate was 10 °C/min. The degree of crystallinity (*X*_c_) of the samples were calculated according to Equation (1) [14]:

(1) $$X_{c}[\%]=\frac{\Delta H_{f}}{\Delta H_{f}^{100\%}}\times 100$$

where $\Delta H_{f}$ and $\Delta H_{f}^{100\%}$ are the melting enthalpy determined by the peak area in the DSC curve and a 100% crystallized PVDF, i.e., 104.7 J/g, respectively.

The two-dimensional (2D) wide-angle X-ray diffraction (WAXD) patterns of the prepared NF thin films were measured using a diffractometer (Rigaku NANO-Viewer, Tokyo, Japan) with monochromic Cu-Kα radiation and recorded on an imaging plate. The axial direction of the aligned NF thin films was set along the meridional direction of the imaging plate. The degree of the preferred crystal orientation (*H*) was expressed by evaluating the full width at half-maximum (FWHM) intensities of the peaks corresponding to the arcs in the WAXD images using Equation (2) [14]:

(2) $$H=\frac{180-w}{180}$$

where *w* is the azimuthal FWHM.

The transverse piezoelectric constant *d*_31_ of the poled NF thin films was measured using a dynamic mechanical analysis (Rheolograph Solid, Toyo Seiki Seisakusyo, Tokyo, Japan). The frequency of dynamic force varied from 1 to 100 Hz at 25 ± 1 °C. The static load and initial strain were 300 g and 0.1%, respectively. The output voltage was measured using an oscilloscope (TDS 2024B, Tektronix, Beaverton, OR, USA) attached to the system. The piezoelectric response of a single as-spun NF on a Si wafer was characterized using an environmentally controlled atomic force microscope (AFM 5300E, Hitachi High-Tech Corporation, Tokyo, Japan) in dynamic force mode (DFM) and piezo response mode (PRM). Rh-coated cantilevers with a spring constant of approximately 15 N/m and a free resonance frequency of 110–150 kHz (SI-DF20-R, Hitachi High-Tech Corporation, Tokyo, Japan) were used. All measurements were performed at 25 ± 1 °C.

## 3. Results and Discussion

### 3.1. Preparation and Characterization of Aligned PVDF Nanofibers

**Figure 2:**
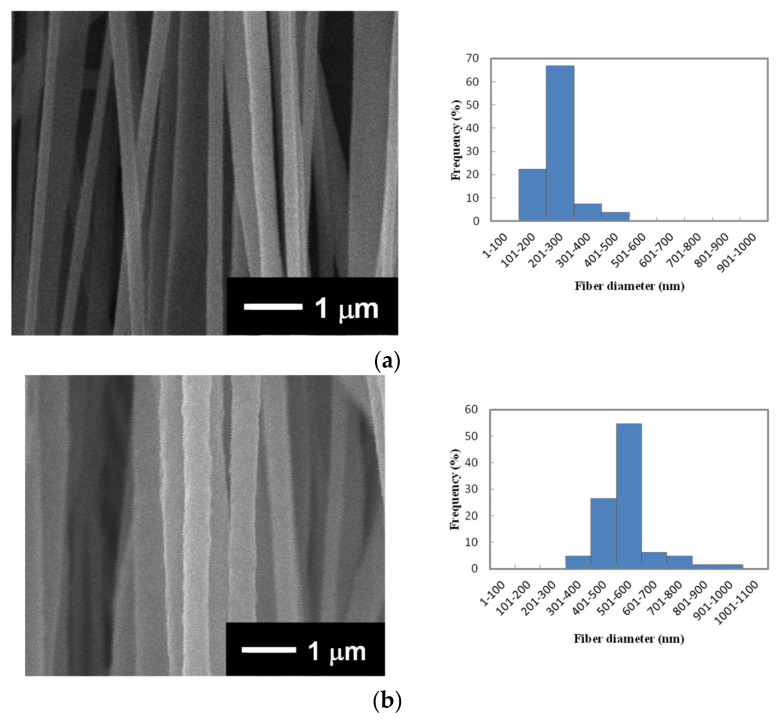

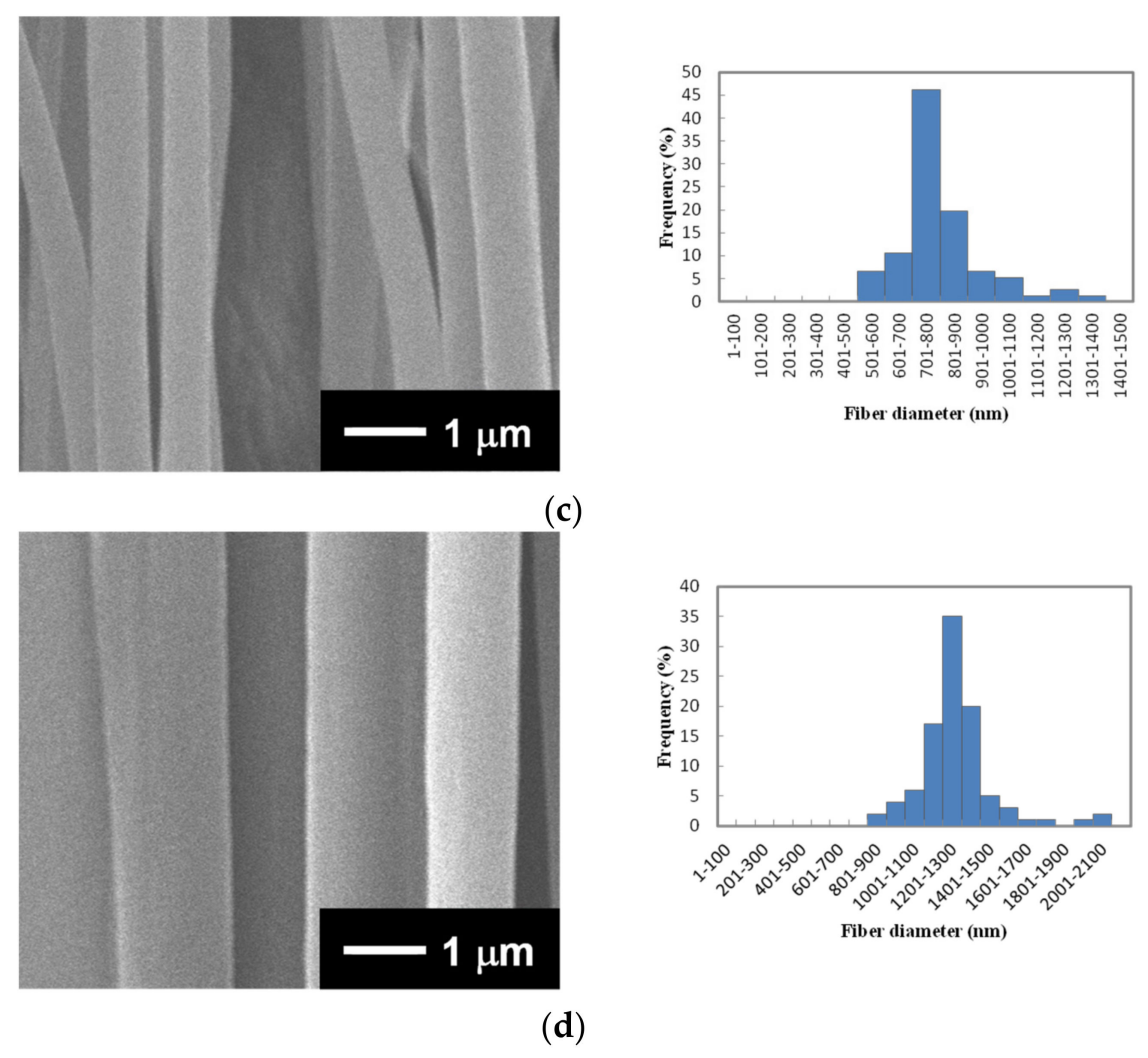
Surface SEM images and their fiber diameter distributions determined by means of SEM image analysis for aligned PVDF NF thin films fabricated from (**a**) NF1, (**b**) NF2, (**c**) NF3, and (**d**) NF4 solutions listed in Table 1.

Figure 2 shows the surface SEM images and fiber diameter distribution of the aligned PVDF NF thin films. By examining the composition of the spinning solutions listed in Table 1, bead-free and well-aligned NFs, with average diameters (*D*) of 228 ± 48, 538 ± 114, 823 ± 191, and 1315 ± 364 nm, were obtained from PVDF/DMAc or PVDF/DMAc–acetone (2:1 *v*/*v*) solutions. The four types of bead-free aligned PVDF NF thin films prepared from the spinning solutions S1, S2, S3, and S4 listed in Table 1 are annotated as NF1, NF2, NF3, and NF4, respectively. For the PVDF/DMAc solutions (S1–S3), as the concentration of the spinning solution increased from 20 to 28 wt%, the *D* increased from 228 to 823 nm. This was due to the increase in the solution viscosity (Table 1). On the other hand, the addition of acetone with a low boiling point (b.p.) of 56 °C to DMAc with a high b.p. of 165 °C resulted in the formation of thicker NFs with a *D* of 1315 nm, caused by accelerated rapid solidification during electrospinning using the 22 wt% PVDF/DMAc–acetone solution (S4).

**Figure 3:**
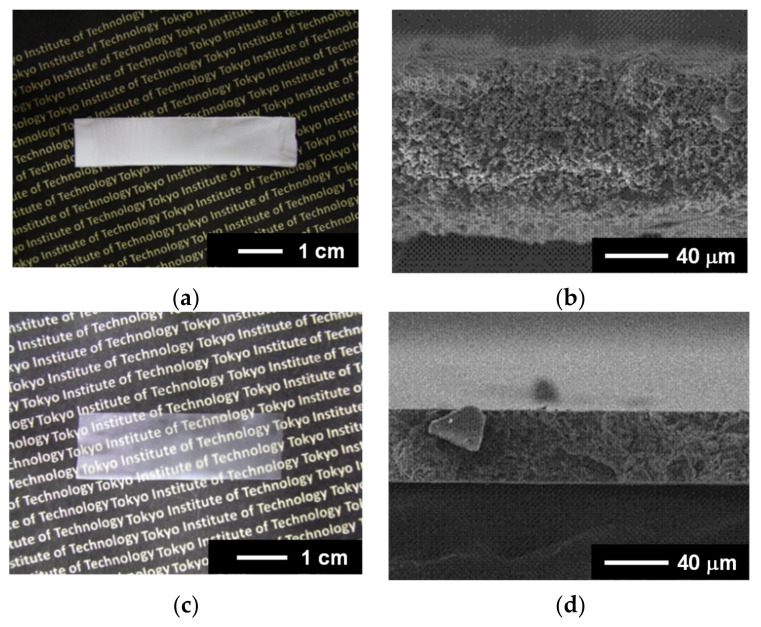
(**a**) Typical photograph and (**b**) cross-sectional SEM image of as-spun thin films (NF1). (**c**) Typical photograph and (**d**) cross-sectional SEM image of hot-pressed NF thin films (NF1).

A typical photograph and SEM images of the prepared PVDF NF thin films are shown in Figure 3. The hot-press changed the appearance of the NF thin films from white to transparent (Figure 3a,c). The SEM images showed that a defect-free, highly packed, dense microstructure was formed by the hot-press (Figure 3b,d). This microstructure is consistent with the transparency of the hot-pressed NF thin films.

**Figure 4:**
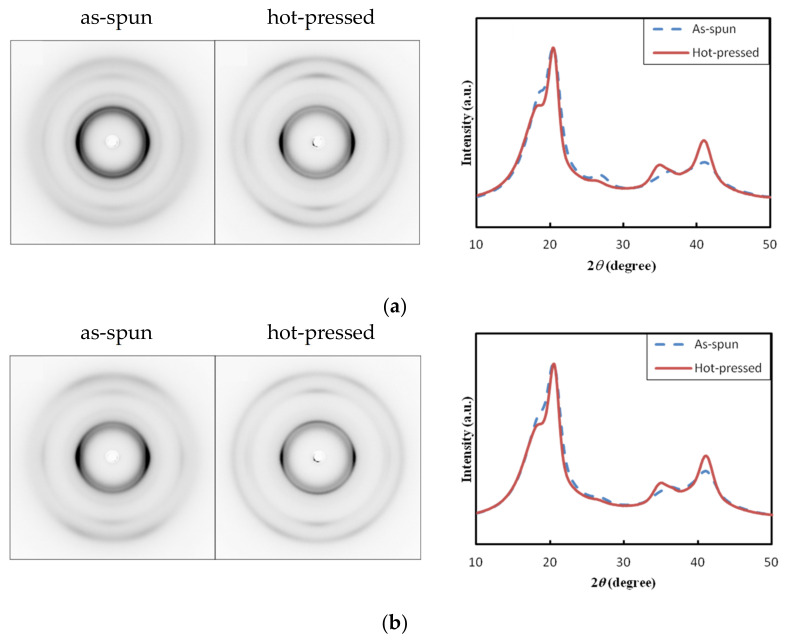

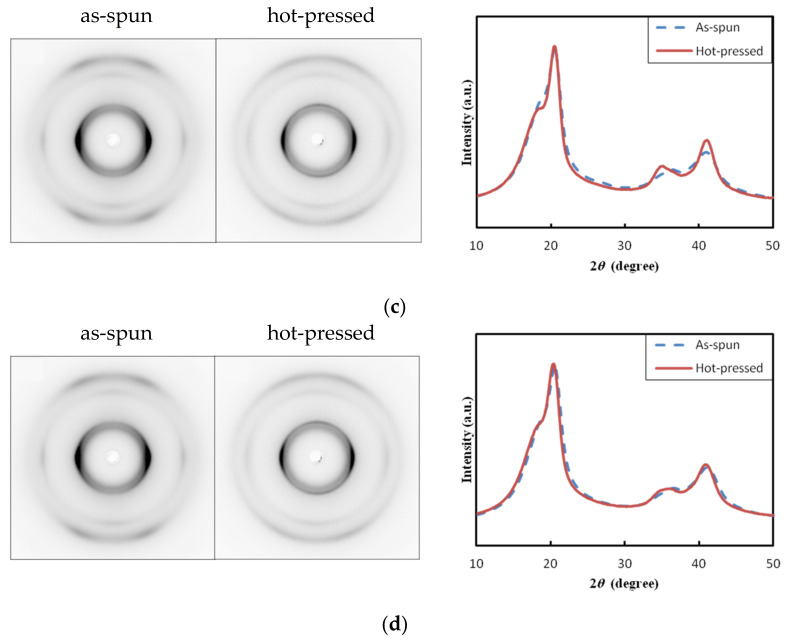
Typical 2D WAXD patterns and 1D profiles of the aligned PVDF NF sheets before and after hot-pressing: (**a**) NF1, (**b**) NF2, (**c**) NF3, and (**d**) NF4. The meridian direction is the fiber axis direction.

Wide-angle X-ray diffraction (WAXD) measurements were performed to investigate the internal structures of the aligned PVDF NF thin films. Figure 4 shows the 2D-WAXD patterns and 1D profiles over the whole azimuthal angles of the NF thin films before and after hot-pressing. For all of the NF thin films, a strong peak was observed at 2*θ* = 20.3°, which is assigned to the (110/200) reflection of the β-phase crystal of PVDF [19,28,34]. In addition, another β-phase peak appeared at 2*θ* = 36°, which is assigned to the (020/101) reflection [28]. On the other hand, an additional shoulder peak was observed at 2*θ* = 18.4°, which is assigned to the reflection of the α-phase crystal of PVDF [19,28]. These results indicate that the prepared NF thin films mainly contained β-phase crystals.

**Figure 5:**
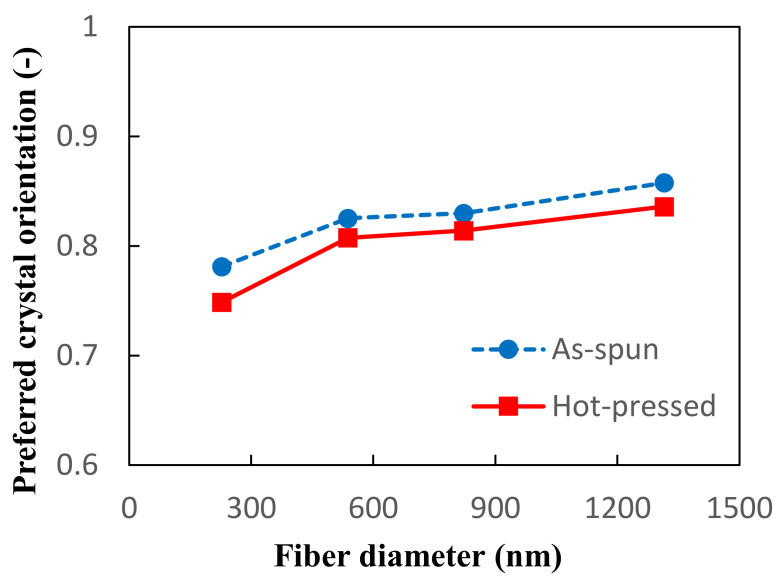
The effect of the fiber diameter on the preferred crystal orientation (*H*) of the (110/200) reflection for the as-spun and hot-pressed PVDF NF thin films.

In Figure 4, the peak at 2*θ* = 20.3° appears as an arc centered on the equator. The azimuthal WAXD profiles of the (110/200) reflection at 2*θ* = 20.3° are shown in Figure S1 of the Supplementary Materials. Sharp peaks were observed at the azimuthal angles near 90 and 270°, indicating that the β-phase crystals were preferentially oriented along the fiber axis. Figure 5 shows the fiber diameter dependence of the *H* value of the (110/200) reflection (i.e., β-phase crystal orientation) for the as-spun and hot-pressed NF thin films. The *H* value increased with an increase in the fiber diameter. This could be explained by shear force, which is based on the intrinsic solution viscosity and/or the increased viscosity induced by solvent evaporation during electrospinning, which enhanced the orientation of the β-phase crystals inside the NFs. For all of the NF thin films, the orientation index was slightly decreased by the hot-press. This was due to a small deterioration in the fiber alignment during hot-pressing.

The typical DSC curves and thermal properties of the as-spun and hot-pressed PVDF NF thin films are presented in Figure S2 and Table 2, respectively. All NF thin films exhibited similar melting points (*T*_m_). The degree of crystallinity (*X*_c_) values obtained from the DSC curves were approximately 50% for all of the NF thin films. Electrospinning substantially increased the *X*_c_ value from 44 to 49–53%, whereas the effect of hot-pressing on the *X*_c_ value was not substantial.

### 3.2. Piezoelectric Properties of PVDF Nanofiber Thin Films

**Figure 6:**
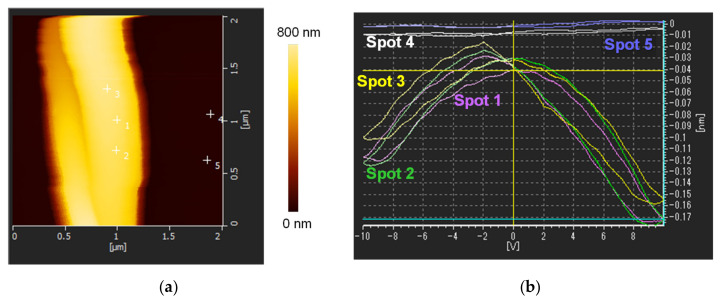
(**a**) Typical AFM topology images of single PVDF NFs with a diameter of ≈500 nm (NF2, magnification area of 2000 nm × 2000 nm). (**b**) Local butterfly loops of the single PVDF NF at different measurement spots. The corresponding spots are shown in (**a**). For the loops at spots 4 and 5, responses could not be detected. A sweeping DC bias (frequency = 5 kHz) in the range of ±10 V was applied at the tip to induce a piezoelectric response.

As shown in Figure 6, the prepared as-spun single PVDF NFs showed a piezoelectric response. The longitudinal piezoelectric coefficient *d*_33_ of 13.1 pC/N for NF2, obtained from the local amplitude–DC bias voltage loops (so-called butterfly loops) in Figure 6b, corresponds to the reported *d*_33_ values for the single electrospun PVDF NFs (15–25 pC/N) [27,28]. Nevertheless, we could not measure a substantial piezoelectric response of the hot-pressed PVDF NF thin films. One possibility is that the relaxation of the β-phase crystal orientation in the NFs was caused by the heat during hot-pressing.

**Figure 7:**
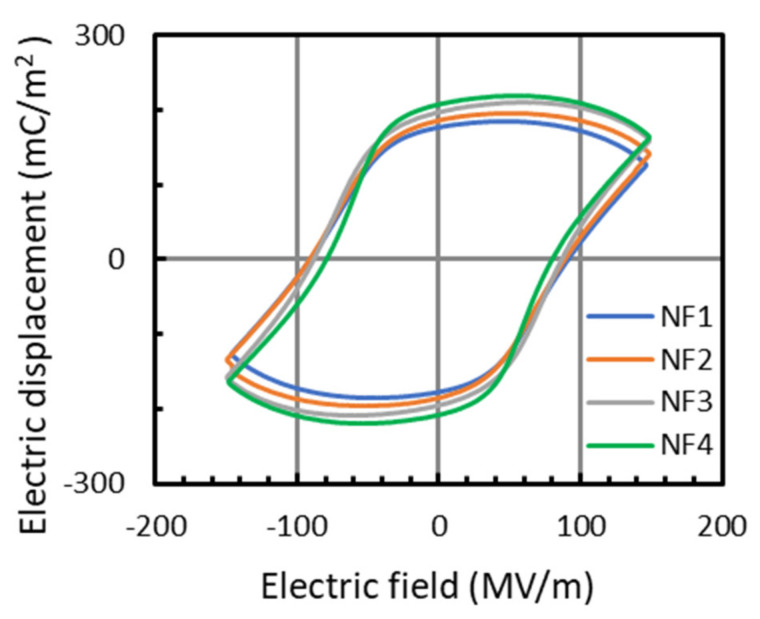
*D*-*E* hysteresis curves of hot-pressed PVDF NF thin films at 40 °C.

To improve the piezoelectricity of the hot-pressed PVDF NF thin films, a poling treatment was carried out. Herein, we used the PVDF NF thin films with a similar thickness of approximately 35–40 μm for poling (see Figure 3; the thickness of the thin films depends on that of the as-spun aligned NF sheets and can be tuned by adjusting the duration of electrospinning). Figure 7 shows the electric displacement–electric field (*D*-*E*) curves of the NF thin films. All of the samples exhibited typical hysteresis curves due to polarization reversal, indicating that the dipoles inside the PVDF NFs were aligned by poling. The residual polarization increased with an increasing fiber diameter.

**Figure 8:**
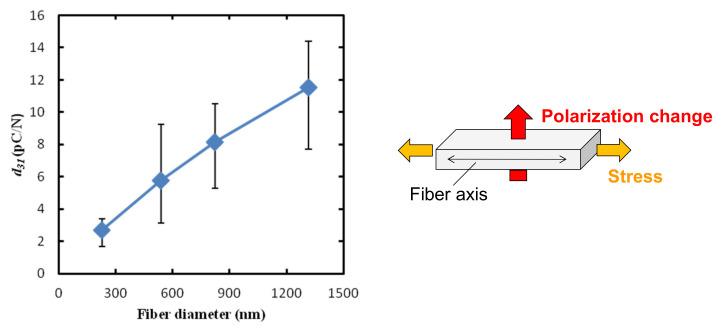
Effect of fiber diameter on transverse piezoelectric coefficient (*d*_31_) at 13 Hz of poled PVDF NF thin films.

After the poling of the NF thin films, the transverse piezoelectric coefficient, *d*_31_, which is the induced polarization normal to the film surface per unit stress applied in the surface direction, was measured. Herein, a constant strain of 0.1% and a sinusoidal force of 13 Hz were applied parallel to the fiber axis of the NF thin films. Figure 8 shows the effect of the fiber diameter on the *d*_31_ values. The *d*_31_ values increased with an increase in the fiber diameter, showing a similar trend as that of the fiber diameter dependence of the preferred crystal orientation (*H*) of the β-phase crystals (Figure 5). The obtained maximum *d*_31_ value of ~12 pC/N is comparable to that of the poled melt-spun PVDF NF (~17.1 pC/N) [35], and it is superior to that of the poled PVDF film under similar conditions (~7 pC/N) [36], and approximately three orders of magnitude larger than those of the near-field electrospun PVDF NF film (0.08~0.10 pC/N) and the electrospun aligned PVDF NF film without successive poling (0.002~0.003 pC/N) [24].

**Figure 9:**
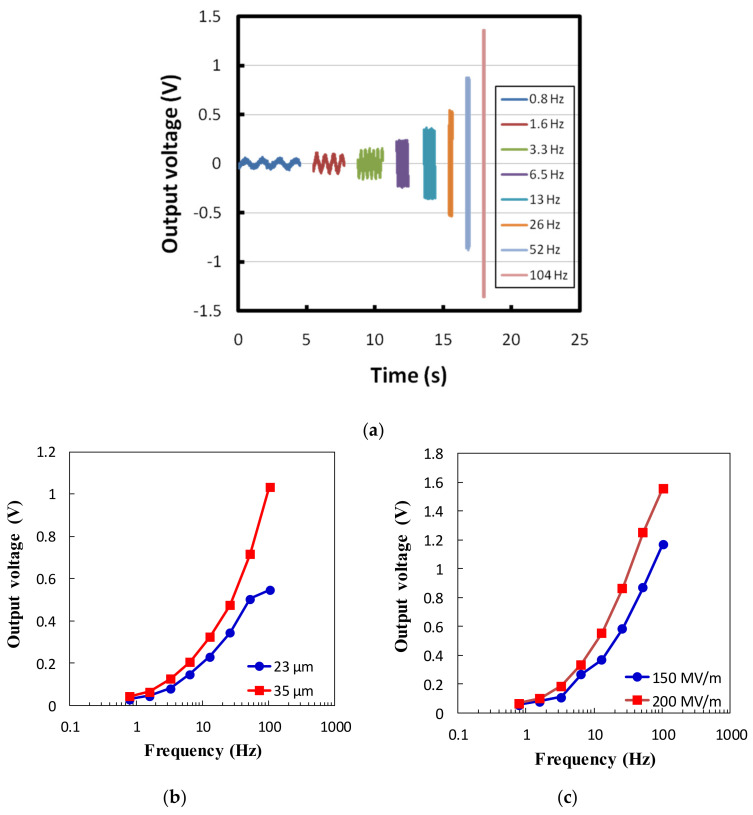
(**a**) Typical actual voltage outputs of the poled PVDF NF thin film under a sinusoidal mechanical stimulation (NF4, film thickness = 25 μm; poling electric field = 150 MV/m; working area = 40 mm^2^; strain = 0.1%). (**b**) The effect of the thin film thickness on the output voltage (NF2, poling electric field = 150 MV/m; working area = 40 mm^2^). (**c**) The effect of the applied electric field during poling on the output voltage (NF2, film thickness = 30 μm; working area = 40 mm^2^).

Finally, we assessed the output performance of the prepared piezoelectric PVDF NF thin films. Figure 9a shows the typical voltage outputs of the poled NF4 thin film with the highest *d*_31_ value. The output voltage increased with an increase in the frequency of the applied strain because the piezoelectric potential response is proportional to the strain rate [37]. The highest peak-to-peak voltage (*V*_pp_) achieved was 2.7 V at 104 Hz for a working area of 40 mm^2^. In principle, it is not possible to directly compare the *V*_pp_ value for samples with different working areas and measuring conditions, but for reference, Zaarour et al. reported that an aligned PVDF NF-based PENG generates ~2.8 V with a working area of 15 cm^2^ and thickness of 100 μm at a frequency of 5 Hz (peak force: 10 N) [31].

To improve the performance, we changed the thickness of the PVDF NF thin films and the applied electric field during the poling treatment; the effects are shown in Figure 9b and Figure 9c, respectively. The increase in both the thickness and the applied electric field results in higher output performance. With an increase in the film thickness, the total amount of the dipoles contained in the working area of the film also increased, consequently generating a higher output voltage. On the other hand, the dipole moments inside the NFs were more oriented under the higher electric field during the poling treatment [38]. These results provide promising insights into the approaches for improving the output performance of piezoelectric polymer NF films.

## 4. Conclusions

In this study, piezoelectric thin films composed of densely packed, uniaxially aligned PVDF NFs with diameters ranging from 228 to 1315 nm were prepared by means of electrospinning with a rotating collector and successive hot-pressing and poling. With an increase in the fiber diameter, the β-phase crystal orientation inside the NFs increased, improving the *d*_31_ value of the thin films. The highest *d_31_* value achieved was 12 pC/N for the thin film composed of the thickest NFs (NF4, *D* = 1315 nm) poled under an applied electric field of 150 MV/m. The maximum *V*_p-p_ value of the prepared thin films that was achieved was 2.7 V at 104 Hz. At present, we have not accomplished the optimization of the piezoelectric properties of densely packed, uniaxially aligned PVDF NF thin films. A better performance can be achieved by further optimizing the molecular orientation during electrospinning, increasing the film thickness, and/or increasing the applied electric field during the poling treatment. Some of these approaches are currently being tested, and the results will be reported. To the best of our knowledge, this is the first report on the relationship between the diameter of aligned PVDF NFs, their internal β-phase crystal orientation, and their piezoelectric properties. The obtained results provide a basis for the study of other piezoelectric polymer NFs. We believe that transparent, flexible, piezoelectric, and aligned PVDF NF thin films can be utilized in various IoT applications including in sensing and energy harvesting devices (e.g., implantable biosensors and wearable and portable electronic devices).

## Figures and Tables

**Table 1:** Compositions and viscosities of spinning solutions.

Solution	PVDF [wt%]	DMAc [wt%]	Acetone [wt%]	Viscosity [Pa s]
S1	20	80	―	0.95
S2	25	75	―	2.1
S3	28	72	―	2.5
S4	22	35	43	1.4

**Table 2:** Thermal properties of aligned PVDF NF thin films.

Sample	Fiber Diameter	*T*_m_ [°C]		*X*_c_ [%]	
	[nm]	As-Spun	Hot-Pressed	As-Spun	Hot-Pressed
NF1	228 ± 48	178	180	51	51
NF2	538 ± 114	180	179	51	53
NF3	823 ± 191	179	177	49	50
NF4	1315 ± 364	178	178	52	52
As-received PVDF ^a^	N.A.		―	44	―

^a^ The as-received PVDF was the pellet used to prepare the spinning solutions.

## Data Availability

The data are available upon request from the authors.

## Supplementary Materials

The following supporting information can be downloaded at https://www.mdpi.com/article/10.3390/nano14060491/s1, Figure S1: Azimuthal WAXD profiles of the (110/200) reflection at 2*θ* = 20.3° for the as-spun and hot-pressed PVDF NF thin films; Figure S2: DSC thermograms for the as-spun and hot-pressed PVDF NF thin films.
