# Supplementary material for: Structure–Piezoelectric Property Relationships of Thin Films Composed of Electrospun Aligned Poly(vinylidene fluoride) Nanofibers
Source: Nanomaterials (Basel). 2024 Mar 8;14(6):491. doi: 10.3390/nano14060491 (PMC10976099; doi:10.3390/nano14060491)
Supplement: Supplementary file 1 [file nanomaterials-14-00491-s001.zip › nanomaterials-2827697-supplementary.pdf]

Supplementary Material

Structure–Piezoelectric Property Relationships of Thin Films Composed of Electrospun Aligned Poly(vinylidene fluoride) Nanofibers

Priangga Perdana Putra, Shuichi Akasaka *, Yuichi Konosu, Shaoling Zhang, Akihiko Tanioka and Hidetoshi Matsumoto *

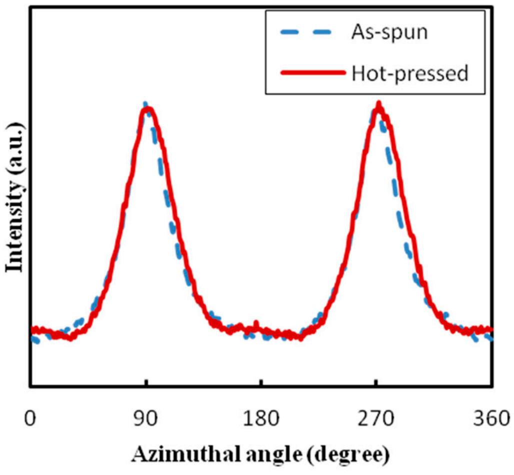

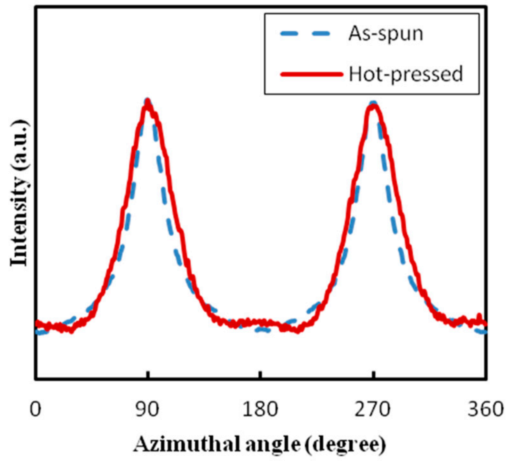

(**a**) (**b**)

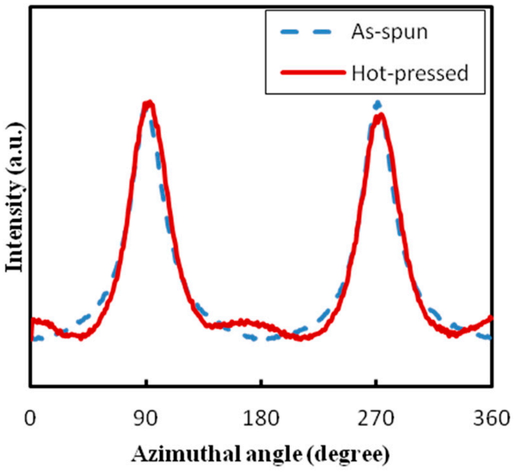

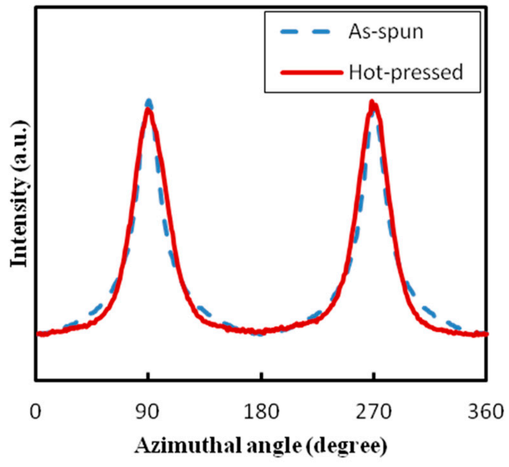

(c) (d)

Figure S1. Azimuthal WAXD profiles of the (110/200) reflection at 2θ = 20.3° for the as-spun and hot-pressed PVDF NF thin films: (a) NF1, (b) NF2, (c) NF3, and (d) NF4.

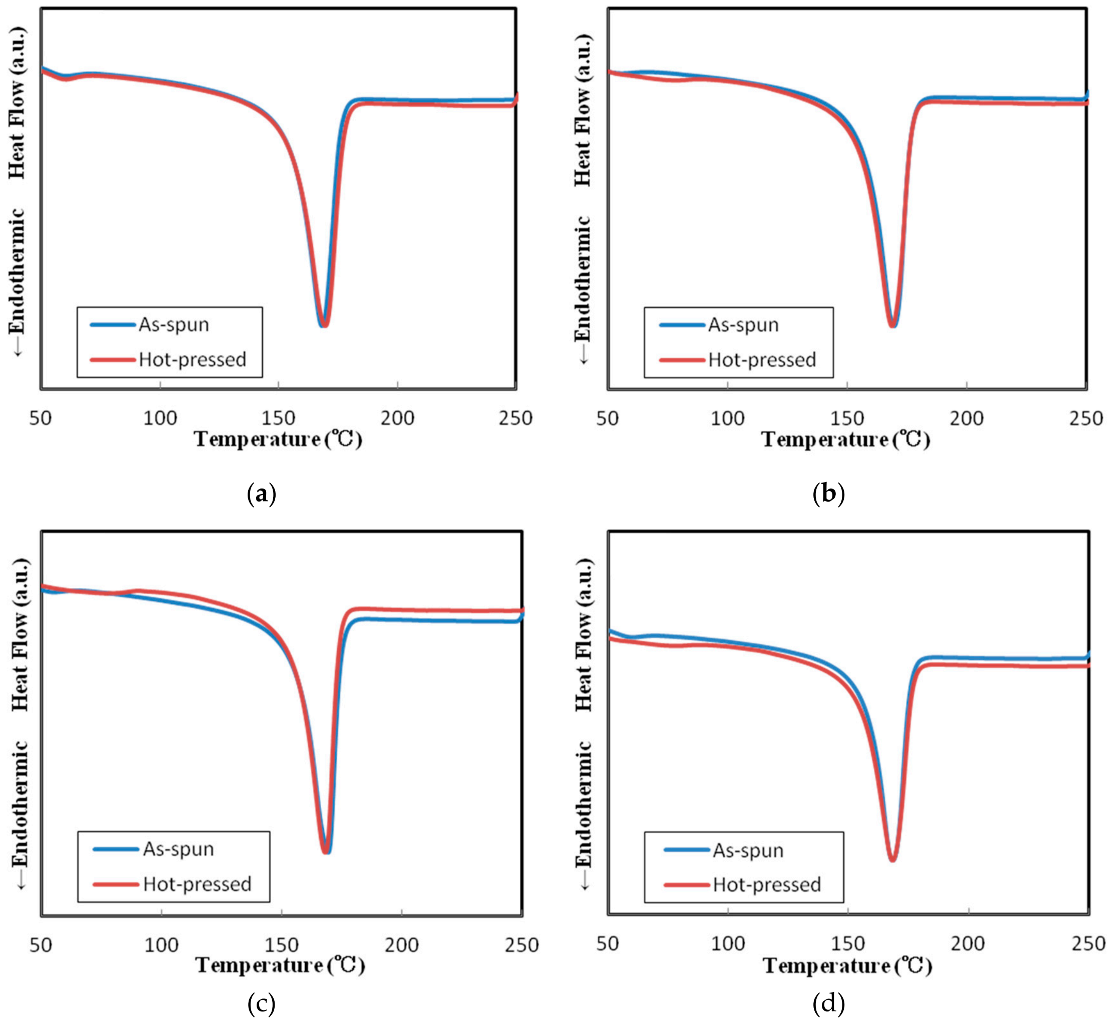

Figure S2. DSC thermograms for the as-spun and hot-pressed PVDF NF thin films. (a) NF1, (b) NF2, (c) NF3, and (d) NF4.
